# Mind Reading?

**Published:** 1873-09

**Authors:** 


### Editorial.

### *Mind Reading?*

Quem Deus vult perdere prius dementat.

"Who sups with the Devil should have a long spoon."

